# Progress in Surgery

**Published:** 1895-07-27

**Authors:** 


### PROGRESS IN SURGERY.

### CEREBRAL SURGERY *(Continued from p. 272).*

**Cerebral Tumours.**—Professor Horsley has lately discussed the differential diagnosis of these growths.[9] He considers that meningitis, abscess, and even uræmic poisoning may precisely imitate cerebral tumour, and lays stress on the fact that in abscess—a condition which he thinks often indistinguishable from tumour—the temperature is often subnormal. Optic neuritis in cases of cerebral tumour he has found most marked on the side of the lesion. Tumours of the frontal lobe appear to produce generalised convulsions, especially of a semi-purposive character, and in the fit the focus which commences the disturbance is that for turning the head and eyes to the opposite side. In the parietal region the convulsions assume the well-known form of Jacksonian epilepsy. In the parieto-occipital region the convulsions will be found in the main to be generalised, and accompanied by ocular deviation and visual aura. The same generalised convulsions are produced by a growth in the occipital lobe, and hemopia is also usually present. Tumours situated in this region may also give rise to cerebellar symptoms from vertical pressure on the tentorium. Even in cases where sometimes the fits are of the typical Jacksonian form, they are generalised at others—often the initial fit is one of general convulsions. A general muscular weakness has been noticed by Horsley in cases of cerebral tumour, a condition which has frequently been confounded with neurasthenia, or has led to the mistaken diagnosis of hysteria. He considers that the view always held by Professor Hitzig, that the so-called motor region is really sensori-motor, is the correct one. The combination of a slight loss of tactile sense with loss of muscular sense he believes to be characteristic of a parietal cortical lesion, and must not, he points out, be confounded with the profound loss of tactile sense which occurs in lesions involving the posterior third of the internal capsule. Paralysis he regards as a late symptom, but convulsions as an early one. He considers it proved that, in a destructive cerebral lesion, the deep reflexes are exaggerated on the opposite side to the lesion; but in destructive cerebellar lesion, on the same side. This point has recently been investigated by Dr. Risien Russell. Professor Horsley thinks that the steady increase—the progressive nature—of the symptoms should always be regarded as the most pathognomonic sign of cerebral tumour. The case of a boy is recorded who had all the symptoms of a parietal lobe tumour, namely, stupor, headache, vomiting, hemiplegia, intense optic neuritis, and apparently Jacksonian fits becoming generalised. The present of nystagmus and reeling gait, however, suggested the presence of a cerebellar growth as well. No tumour was found in the parietal region, but the removal of the trephine disc removed all the symptoms, and the boy recovered. Horsley concludes that release of pressure caused atrophy of the growth, which, he thinks, was cerebellar, with distant pressure symptoms. Dr. Beevor and Mr. Ballance[10] record a case of subcortical cerebral tumour treated by operation. There was a gradual onset of the paralysis, involving successively the right ankle, the knee, and hip, extending after the lapse of seven months to the joints of the right hand, and then to the whole upper extremity; later on speech became affected. The slowly progressive increase of the paralysis the authors regarded as an indication of tumour rather than hæmorrhage, embolism, or thrombosis; and the classical symptoms of intracranial pressure were present—headaches, vomiting, and optic neuritis. They considered that the cortex or subcortical region rather than the internal capsule was the seat of lesion on account of the gradual march of the paralysis, and that the anæsthesia corresponded to the paralysed parts, and was not complete; localisation being imperfect and muscular sense lost. The face was not affected. The absence of fits and cranial tenderness was unlike cortical growth, so that the authors were inclined to localise it as subcortical, and in this position it was found at the operation. The operation was performed in two stages, so as to avoid shock. In the first, a large area of bone was removed, the dura mater remaining unopened, and in the second the growth was removed. The distinction between the edge of the growth and the cerebral tissue was so little marked, that it was doubtful if the whole of the tumour had been removed. The extirpation was carried out by means of a sterilised spoon. She was relieved of her headache, vomiting, double optic neuritis, anæsthesia, and from the greater part of her paralysis; her mental condition, which was very much deteriorated, was restored, the patient regained her bright and cheerful condition. The authors agree with Horsley that in lesions of the cortex, in the so-called motor area, besides Jacksonian epilepsy, and corresponding to the paralysis of the limb segments, there is change of sensibility. Light touches may not be felt, but if they are, they are not properly localised. Loss of muscular sense, so that the patient is not cognisant of the position or of passive movements of the limb, is also present. If a cerebral glioma or sarcoma cannot be removed, the drainage of a cavity in it may relieve all the symptoms. This was the case in a patient under the care of Mr. Ballance and Dr. Gowers. Horsley pointed out some years ago that in cases where a tumour could not be localised, or could not be removed for other causes, the mere removal of a large area of the skull would so reduce intercranial pressure as to remove the symptoms of headache and vomiting, and optic neuritis if present. The authors doubt whether removal of the bone without opening the dura mater would give much relief, and they record an experiment made on a dog to show how little reduction in intracranial capacity removal of even a very large area of the skull will produce. But they place on record a very striking instance of relief of symptoms in a case of tumour, probably of the internal capsule, from removal of bone and incision of the dura mater. They agree with Horsley that two months is the limit for antisyphilitic treatment before undertaking operation. In cases where the typical signs of tumour are absent, and where the fits always begin with the same localised warnings and are attended with loss of consciousness, the authors consider that no operation is advisable unless the paralysis which follows the fits is permanent, or unless the fits occur very frequently; but they consider that any combination of the type symptoms—headache, purposeless vomiting, optie neuritis, with localised fits, would render operation advisable. A case presenting the classical symptoms of cerebral tumour, in which a large area of the skull was removed for the relief of intracranial pressure with marked success, is recorded by Dr. Michell Clarke and Mr. Charles Morton.[11] There were no localising symptoms, and treatment by iodide failed. The headache, which was very intense, was removed by the operation, and optic neuritis, which was present at the time of operation, slowly cleared up, leaving very little atrophy and hardly any impairment of vision. The dura mater was not opened. Mr Morton refers to other cases, published elsewhere, in which great relief was obtained without opening the dura mater. An examination of 100 consecutive post-mortem records of cerebral tumours by Dr. Hale White[12] shows that only ten could have been removed by operation, the growth in the other cases being too deeply seated, multiple, or situated in the cerebellum. A case is reported[13] in which the presence of Jacksonian epilepsy led to exploration of the cortex in the motor area, but nothing abnormal was discovered. Stupor and optic neuritis developed, and the diagnosis of a subcortical growth was confirmed by a second operation, the tumour, which was tubercular, being removed with success.

Dr. Wood and Mr. Cotterell[14] report a case of right hemiplegia with convulsion beginning in the right thumb and fingers. The child fell from the top of a hayrick when a year old. There was no apparent head injury at the time, but in the course of a few weeks the fits began, and the first one left the hemiplegia after it. When three years of age she came under the observation of the authors. The left supraparietal region was distinctly flattened. The condition of paralysis was marked and combined with a certain degree of rigidity. A cyst was found over the left motor area and drained, with recovery from the fits and almost complete recovery from hemiplegia.

[9] Clin. Journ., Feb. 18th, 1895. [10] Brit. Med. Journ., Jan. 5th, 1895. [11] Ibid, April 13th, 1895. [12] Brit. Med. Journ., Dec. 1st, 1894, p. 1,241. [13] Fortschritte de Medicin, No. 13, 1894, and Epitome Brit. Med. Journ., Oct. 27th, 1894. [14] Brit. Med. Journ., Jan. 5th, 1855, p. 10.

